# Hybrid Multi-Dimensional Attention U-Net for Hyperspectral Snapshot Compressive Imaging Reconstruction

**DOI:** 10.3390/e25040649

**Published:** 2023-04-12

**Authors:** Siming Zheng, Mingyu Zhu, Mingliang Chen

**Affiliations:** 1 Computer Network Information Center, Chinese Academy of Sciences, Beijing 100190, China; 2 University of Chinese Academy of Sciences, Beijing 100049, China; 3 School of Engineering, Westlake University, Hangzhou 310024, China; zhumingyu@westlake.edu.cn; 4 Shanghai Institute of Optics and Fine Mechanics, Chinese Academy of Sciences, Shanghai 201800, China; cml2008@siom.ac.cn

**Keywords:** hyperspectral, snapshot compressive imaging, CASSI, compressive sensing

## Abstract

In order to capture the spatial-spectral (x,y,λ) information of the scene, various techniques have been proposed. Different from the widely used scanning-based methods, spectral snapshot compressive imaging (SCI) utilizes the idea of compressive sensing to compressively capture the 3D spatial-spectral data-cube in a single-shot 2D measurement and thus it is efficient, enjoying the advantages of high-speed and low bandwidth. However, *the reconstruction process*, i.e., to retrieve the 3D cube from the 2D measurement, is an ill-posed problem and it is challenging to reconstruct high quality images. Previous works usually use 2D convolutions and preliminary attention to address this challenge. However, these networks and attention do not exactly extract spectral features. On the other hand, 3D convolutions can extract more features in a 3D cube, but increase computational cost significantly. To balance this trade-off, in this paper, we propose a hybrid multi-dimensional attention U-Net (HMDAU-Net) to reconstruct hyperspectral images from the 2D measurement in an end-to-end manner. HMDAU-Net integrates 3D and 2D convolutions in an encoder–decoder structure to fully utilize the abundant spectral information of hyperspectral images with a trade-off between performance and computational cost. Furthermore, *attention gates* are employed to highlight salient features and suppress the noise carried by the skip connections. Our proposed HMDAU-Net achieves superior performance over previous state-of-the-art reconstruction algorithms.

## 1. Introduction

Hyperspectral images contain richer information than common RGB images and are thus widely used in various types of equipment like endoscopic system and remote sensing. To capture the rich spectral information, widely used spectrometers are mostly based on scanning to capture the three-dimensional (3D) spatial-spectral data-cube, i.e., to capture one 2D spatial frame at one wavelength in one shot and then move the next wavelength. The information captured in a 3D data-cube differs from conventional spatial coordinates [1,2], as it includes spectral information in the third dimension. Though high quality hyperspectral images can be obtained, scanning-based techniques are inefficient with respect to capturing dynamic scenes because of accuracy limitations imposed by moving objects or moving devices [3]. Thanks to compressive sensing (CS) [4,5], instead of sampling the spectral data-cube directly, the snapshot compressive-spectral imaging (SCI) [6] system samples the high dimensional data in an indirect manner. In particular, the first designed spectral SCI system, named coded aperture snapshot spectral imaging (CASSI) [7], uses a physical mask (coded aperture) and a disperser to modulate different channels (each channel corresponding to one wavelength) of the hyperspectral image and then captures the modulated data-cube in a snapshot 2D measurement by integrating across the wavelengths.

**Figure 1:**
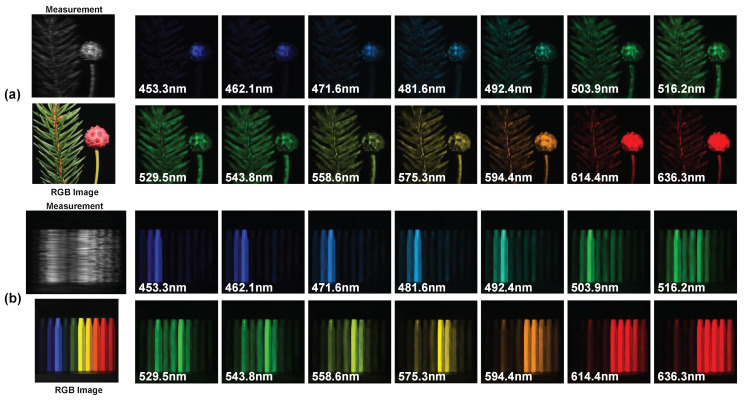
(**a**) The RGB references and reconstructed spectral images of a real measurement captured by [9] with 28 spectral bands (14 are shown here) using our HMDAU-Net. (**b**) One simulated data result (scene 9 in Table 1). The RGB images are shown as a reference.

In this way, a 3D hyperspectral image can be compressed as a 2D measurement (please refer to the left part of Figure 1) and captured by an optical sensor in a short time, thus paving the way for high-speed hyperspectral image sampling [8]. With this high-speed imaging, the data storage and transmission efficiency will be extremely prompted and thus SCI has its promising prospect. After a 2D measurement is acquired, the reconstruction algorithms are employed to recover the 3D spectral data-cube (please refer to the right part of Figure 1).

It has been over 14 years since the first CASSI was built; though different variants of the hardware have been developed [11,12,13], the reconstruction algorithm has been the long-term bottleneck that precludes the wide applications of spectral SCI. Conventionally, the iterative algorithms developed for CS have been used [14,15,16,17], but have been limited by the speed [18] or performance. Fortunately, recent advances in deep learning (DL) open a new window for the inverse problem in imaging [19]. Motivated by this, different DL-based algorithms have been proposed for spectral SCI [9,20,21,22,23,24,25]. However, most existing DL methods borrow the idea from other image restoration problems; for example, both λ-Net [20] and TSA-Net [9] are based on U-Net [26]. These networks usually use 2D convolutional neural networks (CNNs) that ignore the strong correlation among different spectral channels in the data-cube, though some preliminary attention modules are employed. On the other hand, the 3D CNN is able to extract high-dimensional features but suffers from low speed during training and testing.

Bearing these in mind, in this paper, we propose a hybrid multi-dimensional attention U-Net (HMDAU-Net) to reconstruct hyperspectral images from the 2D measurement in an end-to-end manner. HMDAU-Net integrates 3D and 2D convolutions in an encoder–decoder structure to fully utilize the abundant spectral information of hyperspectral images with a trade-off between performance and computational cost. Furthermore, *attention gates* [27] are employed to highlight salient features and suppress the noise carried through the skip connections.

Note that while reconstructing hyperspectral images, we not only need to focus on the spatial resolution but also need to take the spectral resolution into consideration. Though 2D convolutions can capture spatial features well, they lack the ability to effectively investigate the spectral correlation across the third dimension. Hence, we introduce 3D CNN for reconstruction. Due to the greater computational cost of 3D CNN which will increase the inference time, we integrate 3D and 2D CNN for the trade-off of reconstruction fidelity and speed. The utilization of attention gates helps the model to suppress irrelevant regions during training which makes the model pay more attention to the reconstruction details.

### 1.1. Review of the CASSI System

As mentioned above, the key idea of CASSI is to modulate different wavelengths in the spectral data-cube by different weights and then integrate the light to the sensor. The first version of CASSI used a fixed mask and two dispersers to modulate the spatial information over all wavelengths in the spectral cube, termed DD-CASSI [28]; here DD means dual disperser. Following this, the single-disperser (SD) CASSI [7] was developed, which achieves modulation by removing a disperser. Below, we mathematically model the SD-CASSI sensing process.

**Figure 2:**
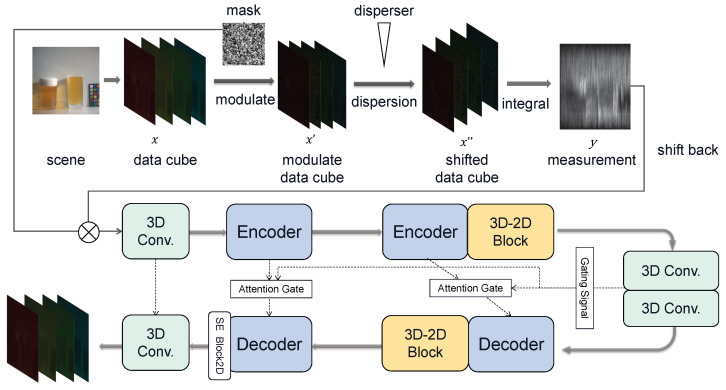
The proposed Hybrid Multi-dimensional Attention U-Net (HMDAU-Net) for CASSI reconstruction. The upper part is an SD-CASSI forward process and the measurement and mask are used as inputs of HMDAU-Net. The network structure shown in the lower part uses the backbone of a two layer U-net, composed of an encoder and a decoder including 3D CNN, 3D Res2Net [31] and 3D maxpooling/transpose 3D CNN. Attention gates [32] and SE (Squeeze-and-Excitation) blocks [33] are employed to extract important correlation information.

Let $X\in R^{W\times H\times B}$ denote the to-be-captured spectral data-cube at the top-left of Figure 2 and $M\in R^{W\times H}$ denote the fixed physical mask, where *W*, *H* and *B* denote the width, height and number of spectral channels, respectively. The spectral data-cube modulated by the coded aperture is $X^{\prime }\left(:,:,b\right)=X\left(:,:,b\right)⊙M$, where $X^{\prime }$ is the same size as X, b=1,2,…,B and ⊙ represents the element-wise multiplication. After propagation through the disperser, each channel of $X^{\prime }$ is shifted along the H-axis according to a liner dispersion d and the respective wavelength. We then use $X^{\prime \prime }\in R^{W\times \tilde{H}\times B}$, where $\tilde{H}=H+d\times \left(B-1\right)$, to denote the shifted cube and assume $\lambda_{c}$ to be the center wavelength which is not shifted when passing through the disperser. We can obtain $X^{\prime \prime }\left(i,j,b\right)=X^{\prime }\left(i,j+d\times \left(\lambda_{b}-\lambda_{c}\right),b\right)$, where (i,j) represents the coordinate system on the plane of the sensor and $\lambda_{b}$ is the wavelength at the *b*-th channel; $d\times (\lambda_{b}-\lambda_{c})$ indicates the spatial shifting of the *b*-th channel. Thus, the 2D SCI measurement $Y\in R^{W\times \tilde{H}}$ we obtain on the detector is a sum over the wavelength dimension of a mask-modulated and later shifted data-cube. It can be modeled as

(1) $$\begin{array}{c} Y=\sum_{b=1}^{B}X^{\prime \prime }\left(:,:,b\right)+N, \end{array}$$

where $N\in R^{W\times \tilde{H}}$ denotes the measurement noise. To facilitate the description of the model, the coding process could be considered as modulating with a shifted mask $\tilde{M}\in R^{W\times \tilde{H}\times B}$ corresponding to different wavelengths and the liner dispersion *d*, i.e., $\tilde{M}\left(i,j,b\right)=M\left(w,h+d\times \left(\lambda_{b}-\lambda_{c}\right)\right)$. Correspondingly, the shifted version $X\in R^{W\times \tilde{H}\times B}$ of the original data-cube is $\tilde{X}\left(i,j,b\right)=X\left(w,h+d\times \left(\lambda_{b}-\lambda_{c}\right),b\right)$. According to this, the 2D measurement Y can be modeled as

(2) $$\begin{array}{c} Y=\sum_{b=1}^{B}\tilde{X}\left(:,:,b\right)⊙\tilde{M}\left(:,:,b\right)+N. \end{array}$$

By vectorizing the spectral data-cube and measurement, that is $x=\mathrm{vec}\left(\tilde{X}\right)\in R^{W\tilde{H}B}$ and $y=\mathrm{vec}\left(Y\right)\in R^{W\tilde{H}}$, this model can be rewritten as

(3) y=Ax+n,

where $A\in R^{W\tilde{H}\times W\tilde{H}B}$ denotes the sensing matrix (coded aperture) which is a concatenation of diagonal matrices, that is $A=[D_{1},\cdots ,D_{B}]$, where $D_{b}=Diag\left(vec\left(\tilde{M}\left(:,:,b\right)\right)\right)$ is the diagonal matrix with $vec(\tilde{M}\left(:,:,b\right))$ as the diagonal elements. Note that A is a very sparse matrix and the theoretical bounds have been developed in [29,30].

After obtaining the measurement y, we will focus on recovering 3D or multi-dimensional information from the 2D measurements, specifically using a novel deep learning network.

### 1.2. Contributions of This Work

In this paper, we propose a new end-to-end deep learning algorithm to reconstruct high quality images for the SD-CASSI system. Our contributions are summarized as follows:

- **Hybrid 3D/2D CNN network**: To balance the performance and computational cost, a hybrid 3D/2D block is employed to reduce parameters. Higher performance is achieved than existing 2D CNN-based algorithms; In addition, the proposed hybrid 3D/2D network shows superiority compared to the pure 3D and 2D counterparts.
- **Wider rather than deeper**: We evaluate that a two layer U-Net has similar performance to a four layer one in CASSI reconstructions.
- Effects of **attention gate** and SE (Squeeze-and-Excitation) block [33] in CASSI are evaluated. Attention gate is implemented to filter the noisy information from U-Net bottleneck and former layers. A simple 2D-CNN SE block is used to focus on important channels.

### 1.3. Related Work

After the first CASSI system [28] was designed, many revised CASSI were proposed. A single disperser CASSI (SD-CASSI) system was designed [7] the following year. Wang et al. [12] designed a Dual-camera CASSI system. Zhang et al. [34] proposed a novel snapshot spectral imaging system that can dynamically capture the spectral images with low computational burden and high light efficiency.

For CASSI reconstruction, the early algorithms are based on iterative optimization algorithms like TwIST [14], GAP-based [15,35] and other algorithms [16,17,18,36,37,38,39]. To promote these iterative algorithms, a deep neural network is inserted into an iteration step as a deep denoiser prior named deep plug-and-play algorithm [40]. Deep unfolding and deep unrolling methods [23,41,42,43,44,45] unfold an iterative algorithm and insert a deep learning network with better performance than common iterative algorithms and maintain their interpretation. The recent work [43] introduced a data-driven prior to exploit both the local and non-local correlations among the spectral image adaptively.

On the other hand, end-to-end deep learning-based algorithms enjoy its high reconstruction speed and excellent performance [46,47,48,49]. Researchers [22,50] proposed a CNN-based method to learn the deep prior externally (dataset) and internally (spatial-spectral constraint of inputs). Meng et al. proposed a TSA-Net [9] to exploit the self-attention mechanism to reconstruct the HSI images by capturing the information across dimensions. A generative adversarial network (GAN) [20] was also introduced in reconstruction.

Real CASSI systems always include noise and thus influence the reconstruction. Zhang et al. [51] modeled the real noise with non-zero mean that generalizes the traditional zero mean noise to characterize the optical imaging principle and boost the reconstruction quality of CASSI. The work [9] found that the shot noise is more suitable for real data training than Gaussian noise as well.

## 2. Proposed Network for CASSI Reconstruction

In this section, we first overview the hybrid multi-dimensional attention U-Net (HMDAU-Net). Following this, different modules of the proposed network are described in detail.

### 2.1. Overall Network Structure

As shown in Figure 2 (lower part), our network consists of a two layer U-Net [26] backbone, 3D–2D hybrid blocks, SE blocks and attention gates. The backbone is a two layer U-Net which is a trimmed version of TSA-Net backbone but without the attention module [9]. The encoder includes 3D CNN, 3D Res2Net and 3D maxpooling and the decoder includes 3D transpose CNN, 3D Res2Net and 3D CNN. The ReLU follows each CNN operation without batch normalization. We remove two layers from the original TSA backbone and change it into a 3D CNN with one initial 3D CNN and one end 3D CNN to match channels. A 2D SE block is employed to set the weight of the feature map and enhance the weight of important ones. Due to the large increase in parameters using cascade 3D CNN like DenseNet [52], we employ a hybrid 2D/3D CNN block named E-HCM [53] to solve our CASSI reconstruction problem. Furthermore, Attention gates [27,32] are implemented in our network to *reduce inessential information among each layer*.

### 2.2. Hybrid 2D/3D CNNs

Hyperspectral images contain abundant information across spectral channels; thus, the reconstruction needs to fully explore this information. Two-dimensional CNN extracts feature maps in each channel but lacks the content and relationship among spectral channels. To address this challenge, 3D CNN for hypterspecral image reconstruction is employed in our network. It has been observed in previous work [54] that a 3D full CNN (3D-FCNN) exploring both spatial context and spectral correlation can achieve excellent results on other applications. Different from 2D convolution, a regular 3D convolution is implemented via 3D kernels and feature maps and thus is capable of investigating correlations across spectral channels. However, 3D CNN generates a large amount of parameters during computing. Some methods use split 3D convolution to reduce parameters (i.e., splitting the filter k×k×k as k×1×1 and 1×k×k) [55] to mitigate this shortcoming. However, redundant information along the spectral dimension will be generated due to the high spatial similarity among spectral channels. This also reduces the learning ability of the model in space, which is extremely important for the reconstruction purpose as considered in our work.

To address this challenge, MCNet [56] was proposed to share the context among 3D and 2D units. A split adjacent spatial and spectral convolution (SAEC) was proposed in [53] to tackle this difficulty. It implements 3D convolution along height–width, spectral-height and spectral-width (i.e., filters are 1×k×k, k×1×k and k×k×1). After reshaping, feature maps go through a few 2D convolution units. This *hybrid 3D/2D CNN module* is dubbed E-HCM. In detail, the 3D unit is employed to analyze the relationship of spectra and either horizontal or vertical direction in space. Since the spectral information is acquired, the feature maps after the 3D unit are reshaped into four dimensions to implement 2D convolution to *further extract the spatial information* in the desired image. Based on the consideration of efficiency and computational cost, we employ this module at the end of encoders and decoders in our HDMAU-Net.

### 2.3. Attention Gate

Attention Gates (AGs) [32] are initially proposed to *capture a sufficiently large receptive field or semantic contextual information* in medical images. The AGs are incorporated into the standard U-Net architecture to highlight salient features that are passed through the skip connections. Information extracted from the coarse scales is used in gating to disambiguate irrelevant and noisy responses in the skip connections.

As shown in Figure 2, the gating signal $g\in R^{F_{g}\times N_{g}}$ is generated via a 3D CNN block, including batch normalization and ReLU. The input feature in the *l*-th layer is $x^{l}\in R^{F_{l}\times N_{l}}$. $N_{g}$ and $N_{l}$ are the sizes of a feature map (i.e., channel×width×height), $N_{g}<N_{l}$, $F_{g}$ and $F_{l}$ correspond to the number of feature maps. *g* and $x^{l}$ are inputs of the attention gate in each layer, which can be represented by:

(4) $$\begin{array}{ccc} & \phi_{g}=\mathrm{upsample}\left(\Omega_{g}\left(g\right)\right),\; & \phi_{x}=\Omega_{x}\left(x^{l}\right), \end{array}$$

(5) $$\begin{array}{ccc} & q_{att}^{l}=\psi \left(\mathrm{ReLU}\left(\phi_{x}+\phi_{g}\right)\right),\; & \alpha_{att}^{l}=\mathrm{sigmoid}\left(q_{att}^{l}\right), \end{array}$$

where Ω(·) and ψ(·) denote linear transformation (e.g., $\Omega \left(u\right)=W_{u}^{T}u+b_{u}$, $b_{u}\in R^{F_{int}\times M}$, $W_{u}^{T}\in R^{\left(F_{l}\times N_{l}\right)\times \left(F_{int}\times M\right)}$) conducted by 1×1×1 3D convolutions. $\phi_{g}$, $\phi_{x}$ and $q_{att}^{l}$$\in R^{F_{int}\times M}$, where $F_{int}$ and *M* are intermediate numbers of a feature map and sizes of a feature map, respectively. Attention coefficient $\alpha_{att}^{l}\in \left[0,1\right]$. When the attention is generated, we multiply it with $x^{l}$ from skip connection and then input into decoder.

Motivated by the attention U-Net [27], the same-scale features from the encoder and decoder can be augmented and combined by attention gates. We firstly use attention gates to boost reconstruction of subtle texture in hyperspectral images and enhance the content of each layer during scale transformation in our HMDAU-Net. The output of AGs is then produced by the decoder with scaling conducted by Res2net and upsampling.

## 3. Experimental Results

We now verify the performance of our proposed HMDAU-Net for CASSI reconstruction, firstly on simulation data and then real data captured via the CASSI system [9]. More results are shown in Appendix A.

### 3.1. Simulated Data

We train our model for simulated data (256×256 measurement on the CAVE [57], 31 spectral images of 256×256×31) and test it on 10 scenes cropped from the KAIST [58] dataset provided by the TSA-Net [9], which adopts spectral interpolation on the simulation data to acquire an image of the 28 channels (ranging from 450 nm to 650 nm) as ground truth. Similar to TSA-Net, we randomly crop the hyperspectral data-cube into a spacial size of 256×256 with 28 channels and then use real mask and shift the data-cube via a 2 pixel step to generate a 256×310 measurement. After shifting it back to a 256×256×28 data-cube, we put it into our network. Three-dimensional CNN need five dimensions to input and thus we unsqueeze it into a batchsize×1×28×256×256 data. The number of 3D feature maps after the first 3D CNN is 32 (the second dimension). After it leaves the last block, we squeeze the data into four dimensions.

#### 3.1.1. Comparison with State-of-the-Art Methods

We compare our proposed reconstruction method with several state-of-the-art (SOTA) methods, including three optimization methods (TwIST [14], GAP-TV [15] and DeSCI [18]), a convolutional autoencoder-based method (AE [58]), a deep unfolding method (HSSP [23]), a GAN-based method (λ-Net [20]) and two end-to-end deep learning methods (U-Net [26] and TSA-Net [9]). AE does not perform as well as in the DD-CASSI system shown in Ref [58] because we use their pre-trained model which differs from our SD-CASSI data scenes, wavelenth distributions and spacial sizes. Other experimental results are from [9]. We use the same training dataset in TSA-Net and 10 scenes for test. We can see that the deep learning-based methods achieve better results and our proposed method is better than the past SOTA algorithm TSA-Net. Specifically, as shown in Table 1, our method outperforms the second best method TSA-Net by 0.6dB in average PSNR and 0.016 in average SSIM.

**Figure 3:**
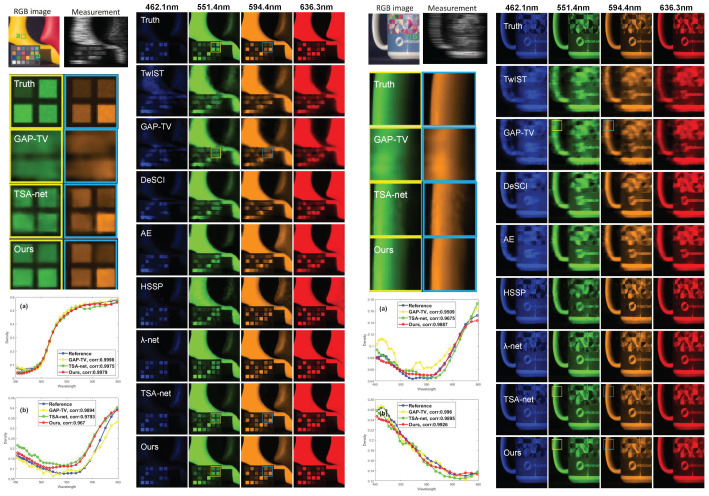
Two reconstructed scenes with four spectral channels using seven methods. We compare the recovered spectra of the selected region (shown with a, b on the RGB images) and spatial details. box “a” and box “b” have been chosen to perform correlation analysis.

Figure 3 plots selected channels (4 out of 28) and spectral curves of the reconstructed images using the methods above. We can obverse that the images reconstructed via the proposed method have clearer texts and stripes. Please notice the letters on the cup and the sharp edges of the color checker. In addition, our method has more accurate spectral density than the other methods.

As depicted in Figure 3, the top-left panel showcases two designated boxes labeled “a” and “b”, accompanied by corresponding reconstructed outcomes and numerical assessments. The assessment procedure involved computing the mean values of boxes “a” and “b” across all wavelengths (each red dot represents an average value of a specific wavelength), followed by correlation analysis of the spectra based on the reference parameter. Our spectral-wise quantitative metrics are shown in the figure and clearly higher than other methods.

#### 3.1.2. Ablation Study

We design several ablation studies to evaluate the effect of different modules in the proposed network. The comparison includes numbers of layers of U-Net backbone, attention gates and hybrid dimensional convolution modules.

To save training time, the experiments in this subsection in simulated data are trained with 16 channels when input into encoder. As shown in Table 2 left, we can observe that a two layer 3D U-Net (using the backbone in TSA-Net and replacing all convolutions by 3D-CNN) has performance similar to a four layer one in CASSI reconstruction. It even achieves 0.22 dB higher PSNR. However, by doubling the feature maps initially input into the encoder, we can see a raise of 0.44 dB. This shows that the assistance of a deeper network is not so distinct and even not beneficial to our SCI reconstruction. Instead, the wider one has much more influence. We find that this may due to the fact that too many downsamplings and upsamplings in spatial and spectral dimensions will cause information loss.

In Table 2 right, we evaluate different modules in our proposed method. Both SE block and attention gates improved our reconstruction results. In particular, SE Block can improve them more (0.25 dB in PSNR) while AGs just edge up a little bit (0.05 dB in PSNR). As we put them together, the promotion is expanded, leading to a 0.27 dB gain in PSNR. This presents the consistency of the two attentions in our reconstruction, without excessively filtering necessary spatial-spectral information.

In Table 3, we implemented different types of convolution in our U-net backbone. Our hybrid backbone uses E-HCM on the second encoder and the first decoder is a two layer U-Net backbone. E-HCM includes three 3D convolution operations and four 2D convolution operations. For the full 3D convolution, we replace the E-HCM by the same number of layer residual blocks (seven layers per module). For full 2D convolution, we replace all 3D convolution operations by 2D and keep the number of layers unchanged. For instance, taking a 2D convolution layer with kernel size *K*, input and output channels $C_{in},C_{out}$ as an example, the number of MACs is $K\times K\times C_{in}\times H_{out}\times W_{out}\times C_{out}$, where $H_{out}$ and $W_{out}$ denote the height and width of the output feature map, respectively. Compared to 2D, 3D improves the PSNR value but significantly increases the computational workload as well. By using our hybrid backbone, we can decrease parameters and computational load to a large extent (40%) in contrast to 3D and even have achieved higher performance than pure 3D and 2D ones. This observation suggests that the pure 3D CNN is not as practical as 2D ones because of the soaring of computational load. However, we can mix it with 2D CNN to make a balance.

### 3.2. Real Data

For the real data captured by the system built in [9], we again borrow the experimental results of other methods. The real data is a 660×714 measurement with 28 wavelengths ranging from 450 nm to 650 nm. It was shifted 54 pixels with respect to dispersion in the column. We train our model again using the real data mask, i.e., 660×660 coded mask and cropped training set. This model is much larger than the simulated one and it takes a huge increase in GPU memory usage (even more than 45 GB for batch size = 1 per batch) and time cost. Thus, we take the advantages of the Automatic Mixed Precision (AMP) module provided by Pytorch to train our model by mixed precision (half precision and single precision real numbers).

**Figure 4:**
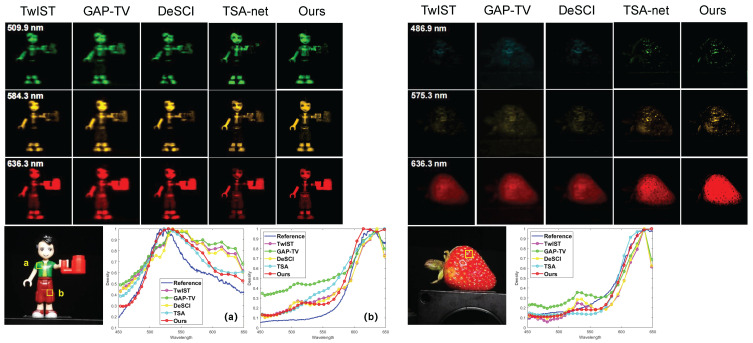
Real data: the reconstructed images of Lego man (**left**) and Strawberry (**right**) for 4 out of 28 spectral channels. area “a” and “b” have been chosen to perform correlation analysis. The spectral curves are shown at the lower part of the figure, the reference curves and RGB images are from [9].

**Figure 5:**
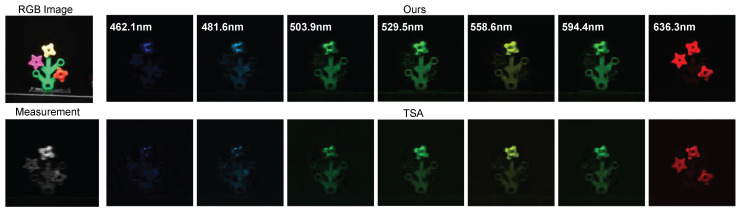
Real data: The RGB references and result images of a real measurement for 7 out of 28 spectral channels reconstructed via TSA and our proposed method.

The reconstruction results of two scenes, Lego and Strawberry are shown in Figure 4, where we plot four reconstructed frames at different wavelengths and spectral density curves to demonstrate the performance of our proposed method. We observe that our result contains more detail in Legoman’s face area because our model produces sharper edges than other models. In the Strawberry testcase, our result has higher spatial resolution in all selected wavelengths. Similar to Figure 3, we attached a visualization of numerical assessment in Figure 4 and the method to obtain such assessment is the same as described above. We observe that our curve (red) is closest to the reference curve (blue) among all other curves.wo more real data results of Plants and Legoplants are shown in Figure 1 and Figure 5 with 14 and 7 selected reconstructed channels, respectively. We selected 7 spectral channels out of 28 as shown in Figure 5. Our model achieves superior reconstruction results in terms of clarity and aesthetics compared to TSA-net. Specifically, our model produces more pointed edges that elevate the overall reconstruction quality.s shown in the plots, our method provides sharper edges and more spacial details such as the hands and clothes of the Lego man. The spectral density curves reveal our method is closer to the ground truth as well.

## 4. Conclusions

We proposed an end-to-end hybrid multi-dimensional attention U-net for hyperspectral snapshot compressive imaging reconstruction. The algorithm employed hybrid 3D/2D convolutions instead of using one of them alone to balance the trade-off of computational cost and performance. Our proposed network achieved superior results over previous end-to-end CNN based algorithms.

One important observation from our experiments is that for SCI reconstruction tasks, it is not necessary that the backbone network (e.g., U-Net) be deep, but it needs to be wider (more kernels in each layer) to provide good results. This may due to the task difference between image reconstruction (to recover details) and image classification (to extract features). We further used the attention gate to extract essential correlations in the spectral data-cube to improve the reconstruction performance in our network.

In addition to spectral SCI reconstruction as shown in this work, we do believe our network can be used in medical images [59], image compression [60], temporal compressive coherent diffraction imaging [61], and video compressive sensing [62,63,64,65,66].

## Figures and Tables

**Table 1:** PSNR in dB (left entry in each cell) and SSIM [10] (right entry in each cell) by different algorithms on 10 scenes in simulation. Best results are shown in **bold**.

Algorithm	TwIST	GAP-TV	DeSCI	AE	U-Net	HSSP	λ-Net	TSA-Net	Ours
Scene1	24.81, 0.730	25.13, 0.724	27.15, 0.794	27.45, 0.813	28.28, 0.822	31.07, 0.852	30.82, 0.880	31.26, 0.887	**32.00, 0.898**
Scene2	19.99, 0.632	20.67, 0.630	22.26, 0.694	22.40, 0.709	24.06, 0.777	26.30, 0.798	26.30, 0.846	26.88, 0.855	**28.00, 0.889**
Scene3	21.14, 0.764	23.19, 0.757	26.56, 0.877	26.47, 0.861	26.02, 0.857	29.00, 0.875	29.42, 0.916	30.03, 0.921	**31.37, 0.939**
Scene4	30.30, 0.874	35.13, 0.870	39.00, 0.965	36.96, 0.950	36.33, 0.877	38.24, 0.926	37.37, 0.962	39.90, 0.964	**40.75, 0.971**
Scene5	21.68, 0.688	22.31, 0.674	24.80, 0.778	24.37, 0.797	25.51, 0.795	27.98, 0.827	27.84, 0.866	28.89, 0.878	**29.08, 0.893**
Scene6	22.16, 0.660	22.90, 0.635	23.55, 0.753	24.64, 0.776	27.97, 0.794	29.16, 0.823	30.69, 0.886	31.30, 0.895	**31.41, 0.919**
Scene7	17.71, 0.694	17.98, 0.670	20.03, 0.772	20.04, 0.786	21.15, 0.799	24.11, 0.851	24.20, 0.875	25.16, 0.887	**25.71, 0.901**
Scene8	22.39, 0.682	23.00, 0.624	20.29, 0.740	24.33, 0.783	26.83, 0.796	27.94, 0.831	28.86, 0.880	**29.69**, 0.887	29.49, **0.900**
Scene9	21.43, 0.729	23.36, 0.717	23.98, 0.818	25.10, 0.793	26.13, 0.804	29.14, 0.822	29.32, 0.902	30.03, 0.903	**31.38, 0.920**
Scene10	22.87, 0.595	23.70, 0.551	25.94, 0.666	24.55, 0.701	25.07, 0.710	26.44, 0.740	27.66, 0.843	**28.32**, 0.848	28.31, **0.859**
Average	22.44, 0.703	23.73, 0.683	25.86, 0.785	25.63, 0.797	26.80, 0.803	28.93, 0.834	29.25, 0.886	30.15, 0.893	**30.75, 0.909**

**Table 2:** Left: The comparisons of using different numbers of layers in the 3D U-net backbone showing average PSNR in dB, SSIM on the 10 scenes. Right: The comparisons of using different modules in our proposed algorithm.

Method	PSNR	SSIM
4 layers-16	29.38	0.892
2 layers-16	29.51	0.886
**2 layers-32**	**29.95**	**0.898**
Our Backbone	29.77	0.888
+SE Block	30.02	0.893
+AGs	29.82	0.887
**+AGs and SE Block**	**30.14**	**0.899**

**Table 3:** The comparisons of using 2D, 3D and hybrid convolution as U-net backbone in our proposed algorithm showing average PSNR, SSIM, model parameters and computational loads on the 10 scenes.

Method	PSNR	SSIM	Parameters	MACs
Our backbone	29.77	0.888	0.446 M	114.9 G
Full 3D Convolution	29.55	0.885	0.700 M	152.9 G
Full 2D Convolution	29.09	0.884	0.270 M	4.6 G

**Figure A1:**
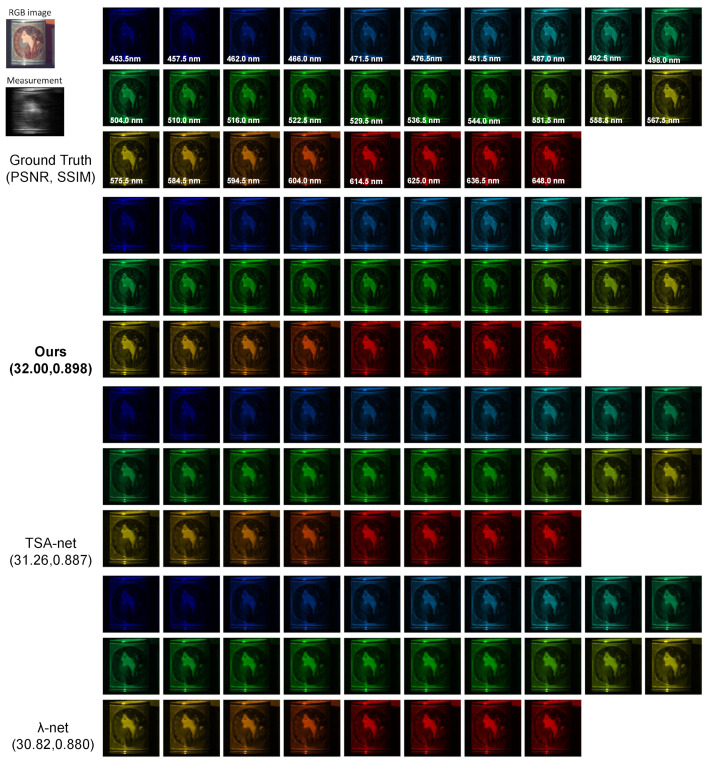
Simulation: RGB image, measurement, ground truth and reconstructed results by Tour proposed method with the TSA-net and λ-net Scene 1. The PSNR in dB and SSIM for the result images are shown in the parenthesis.

**Figure A2:**
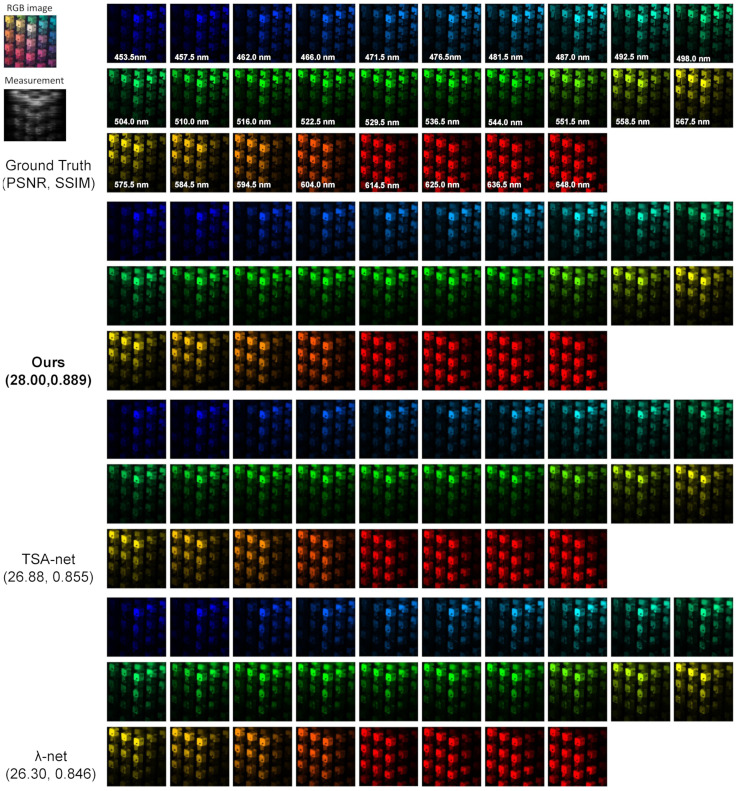
Simulation: RGB image, measurement, ground truth and reconstructed results by Tour proposed method with the TSA-net and λ-net Scene 2. The PSNR in dB and SSIM for the result images are shown in the parenthesis.

**Figure A3:**
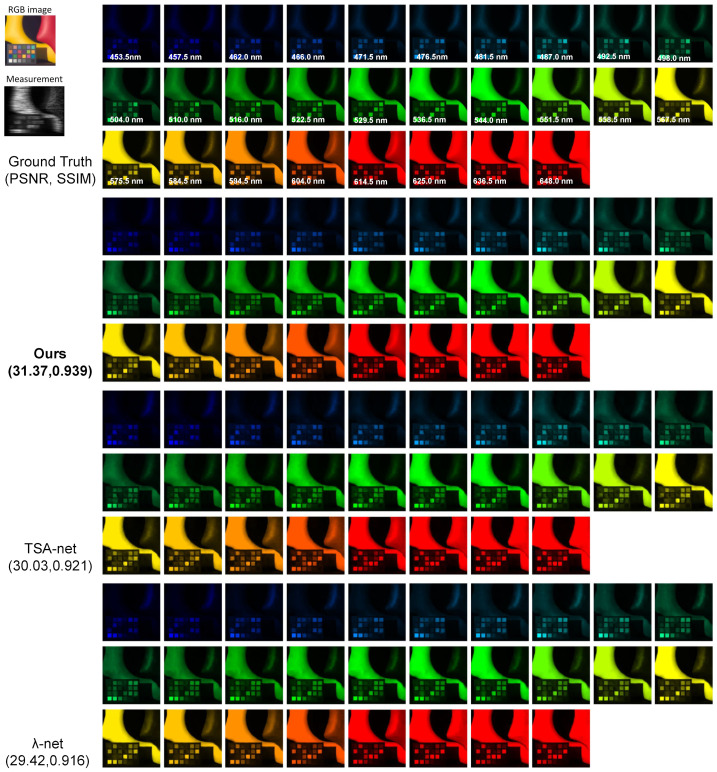
Simulation: RGB image, measurement, ground truth and reconstructed results by Tour proposed method with the TSA-net and λ-net Scene 3. The PSNR in dB and SSIM for the result images are shown in the parenthesis.

**Figure A4:**
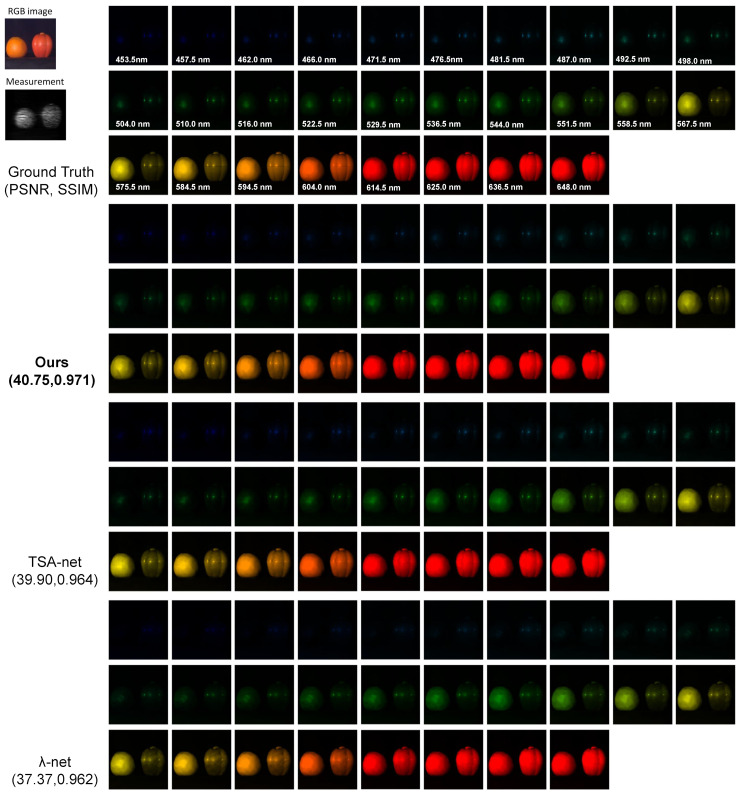
Simulation: RGB image, measurement, ground truth and reconstructed results by Tour proposed method with the TSA-net and λ-net Scene 4. The PSNR in dB and SSIM for the result images are shown in the parenthesis.

**Figure A5:**
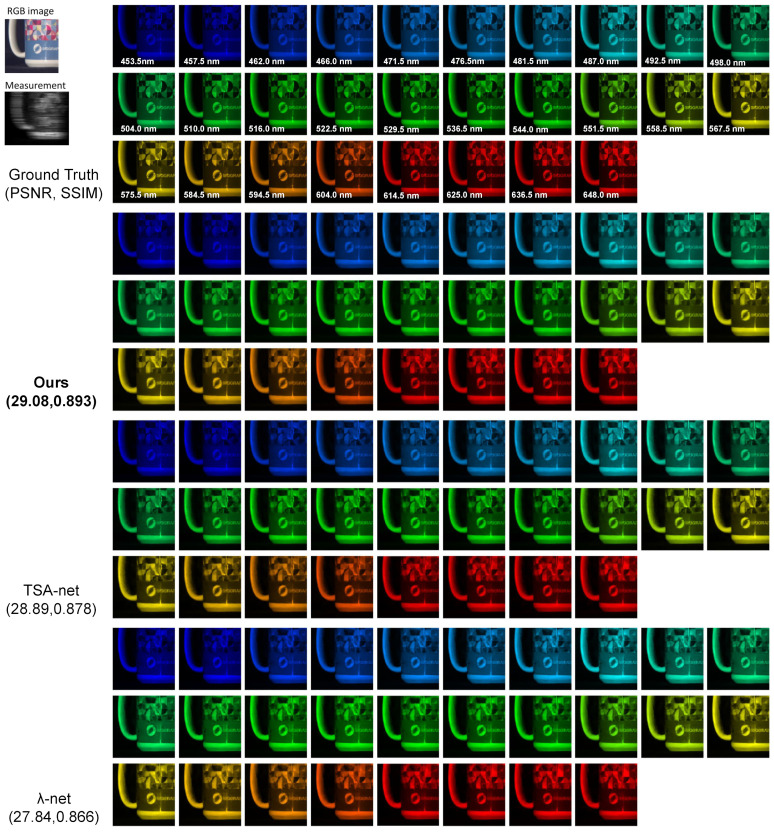
Simulation: RGB image, measurement, ground truth and reconstructed results by Tour proposed method with the TSA-net and λ-net Scene 5. The PSNR in dB and SSIM for the result images are shown in the parenthesis.

**Figure A6:**
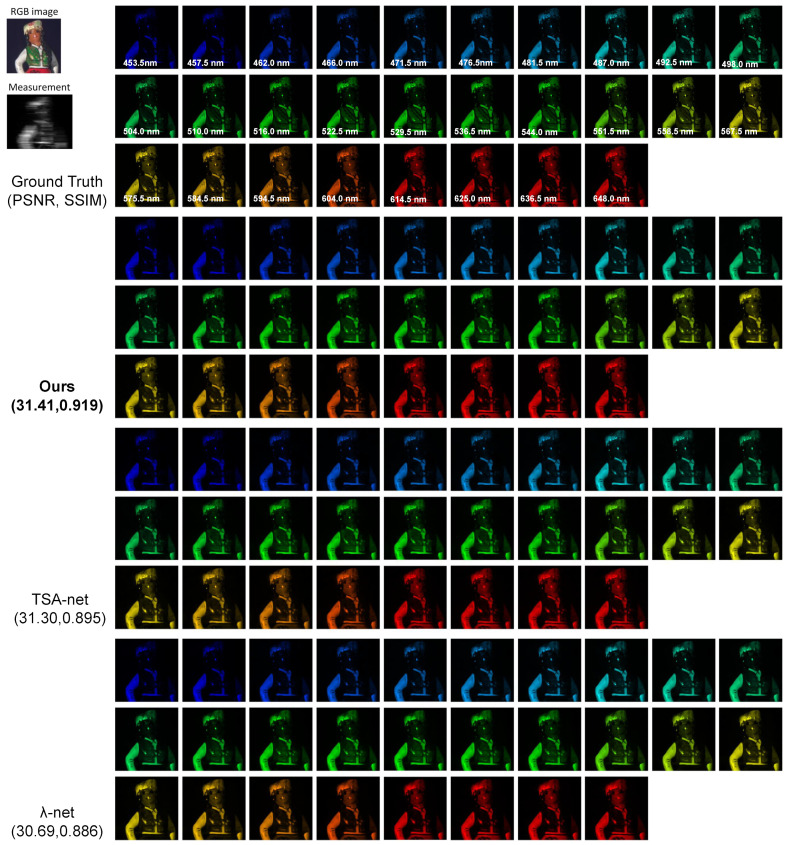
Simulation: RGB image, measurement, ground truth and reconstructed results by Tour proposed method with the TSA-net and λ-net Scene 6. The PSNR in dB and SSIM for the result images are shown in the parenthesis.

**Figure A7:**
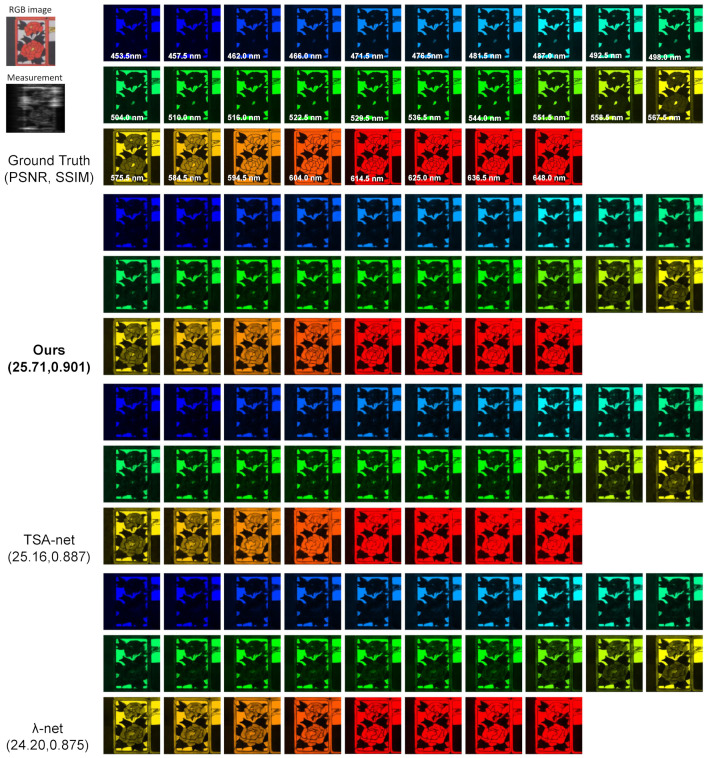
Simulation: RGB image, measurement, ground truth and reconstructed results by Tour proposed method with the TSA-net and λ-net Scene 7. The PSNR in dB and SSIM for the result images are shown in the parenthesis.

**Figure A8:**
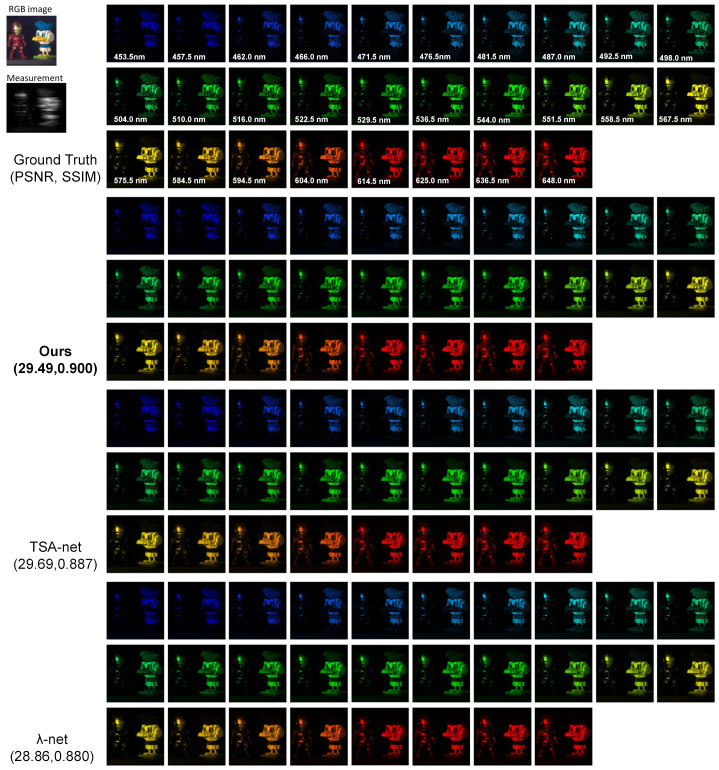
Simulation: RGB image, measurement, ground truth and reconstructed results by Tour proposed method with the TSA-net and λ-net Scene 8. The PSNR in dB and SSIM for the result images are shown in the parenthesis.

**Figure A9:**
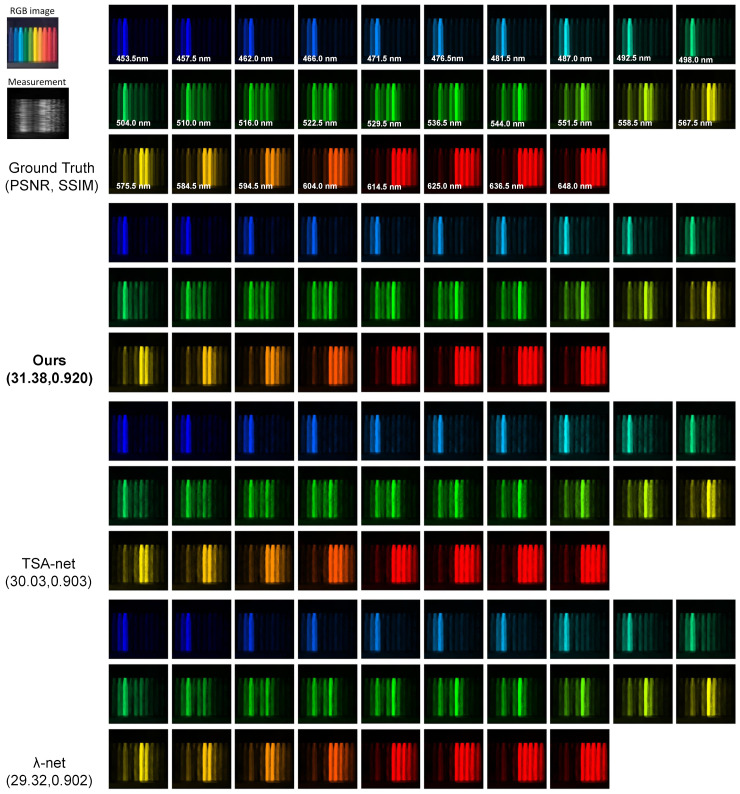
Simulation: RGB image, measurement, ground truth and reconstructed results by Tour proposed method with the TSA-net and λ-net Scene 9. The PSNR in dB and SSIM for the result images are shown in the parenthesis.

**Figure A10:**
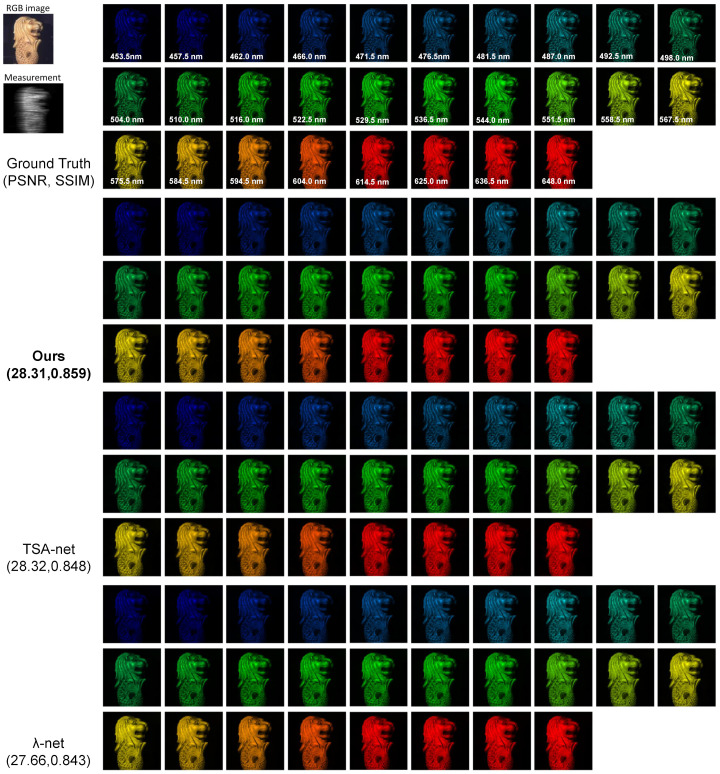
Simulation: RGB image, measurement, ground truth and reconstructed results by Tour proposed method with the TSA-net and λ-net Scene 10. The PSNR in dB and SSIM for the result images are shown in the parenthesis.

**Figure A11:**
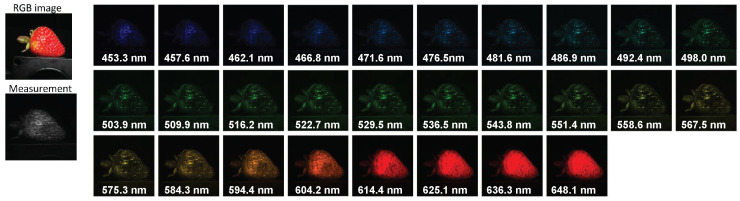
Real data: RGB image, measurement and reconstructed results by our proposed method for scene 1.

**Figure A12:**
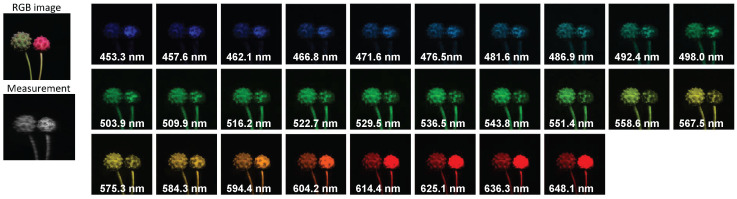
Real data: RGB image, measurement and reconstructed results by our proposed method for scene 2.

**Figure A13:**
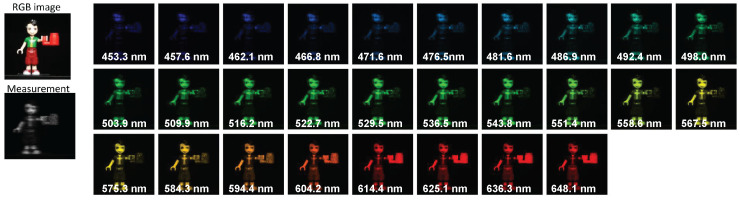
Real data: RGB image, measurement and reconstructed results by our proposed method for scene 3.

**Figure A14:**
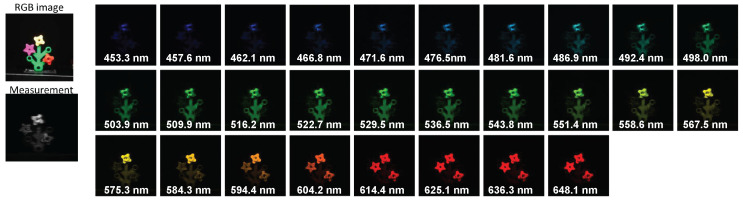
Real data: RGB image, measurement and reconstructed results by our proposed method for scene 4.

## Data Availability

The data that support the plots within these paper and other findings of this study are available from the corresponding authors upon reasonable request.

## Appendix A. Experimental Results

### Appendix A.1. Simulated Data Results

Figure A1, Figure A2, Figure A3, Figure A4, Figure A5, Figure A6, Figure A7, Figure A8, Figure A9 and Figure A10 show the simulation results with 28 spectral channels for 10 scenes from KAIST. Truth, measurements and RGB images are shown for reference. We compare our proposed method with the TSA-net and λ-net algorithms and list the corresponding PSNR and SSIM.

### Appendix A.2. Real Data Results

Figure A11, Figure A12, Figure A13 and Figure A14 show the RGB images, measurements and the reconstructed 28 spectral channels for four real scenes with a size of 660 × 660 pixels captured by real CASSI system.
